# A systematic review of neuroprotective strategies after cardiac arrest: from bench to bedside (part II-comprehensive protection)

**DOI:** 10.1186/2045-9912-4-10

**Published:** 2014-05-20

**Authors:** Lei Huang, Patricia M Applegate, Jason W Gatling, Dustin B Mangus, John Zhang, Richard L Applegate

**Affiliations:** 1 Department of Anesthesiology, Loma Linda University School of Medicine, 11041 Campus Street, Loma Linda, CA, USA; 2 Department of Basic Sciences, Division of Physiology and Anesthesiology, Loma Linda University School of Medicine, 11041 Campus Street, Loma Linda, CA 92354, USA; 3 Department of Cardiology, Loma Linda University School of Medicine, 11201 Benton St, Loma Linda, CA 92354, USA; 4 Department of Neurosurgery, Loma Linda University School of Medicine, 11041 Campus Street, Loma Linda, CA 92354, USA

**Keywords:** Cardiac arrest, Global brain injury, Comprehensive neuroprotection, Model, Pharmaceutical, Hyperbaric oxygen, Hydrogen sulfide, Hydrogen gas

## Abstract

Neurocognitive deficits remain a significant source of morbidity in survivors of cardiac arrest. We conducted a literature review of treatment protocols designed to evaluate neurologic outcome and survival following global cerebral ischemia associated with cardiac arrest. The search was limited to investigational therapies that were implemented either during cardiopulmonary resuscitation or after return of spontaneous circulation in studies that included assessment of impact on neurologic outcome. Given that complex pathophysiology underlies global brain hypoxic ischemia following cardiac arrest, neuroprotective strategies targeting multiple stages of neuropathologic cascades should promise to improve survival and neurologic outcomes in cardiac arrest victims. In Part II of this review, we discuss several approaches that can provide comprehensive protection against global brain injury associated with cardiac arrest, by modulating multiple targets of neuropathologic cascades. Pharmaceutical approaches include adenosine and growth factors/hormones including brain-derived neurotrophic factor, insulin-like growth factor-1 and glycine-proline-glutamate, granulocyte colony stimulating factor and estrogen. Preclinical studies of these showed some benefit but were inconclusive in models of global brain injury involving systemic ischemia. Several medical gases that can mediate neuroprotection have been evaluated in experimental settings. These include hydrogen sulfide, hyperbaric oxygen and molecular hydrogen. Hyperbaric oxygen and molecular hydrogen showed promising results; however, further investigation is required prior to clinical application of these agents in cardiac arrest patients.

## Introduction

According to the 2013 update of Heart Disease and Stroke Statistics from the American Heart Association (AHA), out-of-hospital cardiac arrest (CA) has an overall incidence of 359,400 yearly and a low 9.5% survival rate [1]. Additionally, more than 200,000 adults have in-hospital CA each year and only 23.9% of these patients survive to hospital discharge [1]. For those patients who do survive to hospital discharge, neurologic injury remains a source of significant morbidity. Nearly 70% of patients still have moderate to severe cognitive deficits at three months after CA [2]. The persistence of unfavorable neurologic outcomes, despite advances in cardiopulmonary resuscitation (CPR) prompted AHA to emphasize brain injury in relation to cardiac arrest by proposing “cardiopulmonary-cerebral resuscitation” in its 2000 Guidelines for Cardiopulmonary Resuscitation and Emergency Cardiovascular Care [3]. Therapeutic strategies targeting brain injury after CA are thus an important area for basic and clinical research. Over decades, however, a host of putative neuroprotective strategies failed to demonstrate clinical benefit except for mild therapeutic hypothermia [4]. New neuroprotective approaches are warranted in the setting of global brain injury associated with CA.

The complex series of pathophysiological changes contributing to global brain hypoxic ischemia following CA have been summarized in Part I of this review. Whole body ischemia/reperfusion injury and/or systemic inflammatory responses increase the pathologic complexity of brain injury following CA, which in turn increases the difficulty and complexity in the search for an effective neuroprotective agent. In this context, a specific therapy targeting multiple stages of neuropathology cascades should provide promise to improve survival and neurologic outcomes in CA victims. Indeed, the successful translation of therapeutic hypothermia (TH) to CA patients is attributed to the multiple neuroprotective effects of TH against global brain ischemia [5].

### Literature search method

A literature search was conducted of articles indexed in Medline and published between 1980 and October 2013 using combination of keywords including “brain injury”, “cardiac arrest”, “neuroprotection”, “cerebral protection”, “cardiopulmonary resuscitation”, “global ischemia”, “global cerebral ischemia”, and “global brain ischemia” (Table 1). Bibliographies of relevant articles were cross-referenced for pertinent articles. Articles were selected for review if postulated mechanisms of neuroprotection and some measure of neurologic outcomes were included. Only neuroprotective strategies tested in animal models relevant to global brain ischemia associated with CA were reviewed. Case reports, pediatric studies and articles not written in English were excluded. Studies of therapies administered before CA were not included as our goal was to investigate potential therapies to improve neurologic outcome that can be employed in the clinical setting (during or after CPR). Similarly, the many studies related to neuroprotection from anesthetic agents were not included, as administration of anesthetic agents during or immediately after CPR may be impractical or associated with undesirable hemodynamic effects. Due to the pathophysiologic differences between focal and global cerebral ischemia, the extensive literature regarding neuroprotective strategies in focal cerebral ischemia is acknowledged but not included in this review. In this part, a total of 17 preclinical studies were reviewed. We focus on several strategies that demonstrate comprehensive neuroprotective mechanisms evaluated in the setting of whole body and global brain ischemia associated with CA. Due to the limited numbers of publications in this area, we included pre-clinical studies using animal models of global brain injury involving bilateral carotid occlusion along with systemic hypotension.

**Table 1:** Search terms used to perform literature search

**Database**	**Search terms**
PubMed	Brain injury
	Cardiac arrest
	Neuroprotection
	Neuroprotection
	Cerebral protection
	Cardiopulmonary resuscitation
	Global ischemia
	Gglobal cerebral ischemia
	Global brain ischemia
	These terms were searched in combinations as subject headings and keywords simultaneously.
	Articles were limited to those printed or translated into English

## Review

In addition to global brain ischemia and reperfusion, CA and resuscitation are associated with whole body ischemia/reperfusion injury and/or systemic inflammatory responses. These processes increase the pathological complexity of brain injury following CA. Therapeutic interventions targeting multiple stages of neuropathologic cascades that have been investigated can be separated into PHARMACEUTICAL APPROACHES and GAS-MEDIATOR APPROACHES (Table 2).

**Table 2:** Summary of comprehensive neuroprotective strategies for global cerebral ischemia associated with cardiac arrest

**Therapy**	**Proposed mechanism**	**Study subject**	**Blind**	**Placebo control**	**Random assignment**	**Delivery route**	**Effect:**	**Outcomes evaluated**
							**Positive**	
							**Negative**	
							**Neutral**	
*Pharmaceutical approaches*								
Adenosine [8]	Blockade Ca^2+^ influx [10] Hypothermia [8]	Rats	Not mentioned	Yes	Yes	Intravenous	Positive	Survival, regional blood flow, brain edema, metabolite assay, neurohistopathology, temporalis muscle temperature
BDNF [14]	Up regulate Bcl-2, suppress TNF-alpha, increase IL-10, reduce excitotoxicity, promote neural regeneration and axonal sprouting/synaptogenesis [11]	Rats	Not mentioned	Yes	Not mentioned	Intracerebro-ventricular	Neutral	Survival, neurologic function, neurohistopathology
IGF-1/GPE [19,21]	Anti-apoptosis, modulation of BBB permeability and neuronal excitability [17]	Rats	Not mentioned	Yes	Yes	Intravenous [19]	Positive when combined with TH [19]	Neurologic function [19]
	HIF-1 alpha activation [18]					Intracerebro-ventricular [21]	Short-term: Positive; Long-term: Neutral [21]	Neurohistopathology [18,21]
G-CSF [25,26]	Anti-apoptosis, anti-inflammation and enhance neurogenesis [23]	Rats	Not mentioned	Yes	Yes [25]	Subcutaneous [25]	Positive [25]	Survival, neurologic function, neurohistopathology [25,26]
					Not mentioned [26]	Intracerebro-ventricular [26]	Long-term: Negative [26]	p-STAT3, p-AKT1/2/3 and p-ERK1/2 [25]
Estrogen [30-32]	Promote neuronal survival and neurogenesis [29]	Mice	Not mentioned [30,31]	Yes	Yes	Intravenous [30,31]	Positive	Neurological function [31] Neurohistopathology [30-32]
	Increase expression of SK2 [32]		Yes [32]			Subcutaneous [31,32]		Small-conductance calcium-activated potassium (SK2 and SK3) channel transcripts, electrophysiology [32]
	Reduce excitoxicity [33,34]							
*Gas-mediator approaches*								
H_2_S [41-44]	Increase level of antioxidant glutathione and/or scavenging oxygen species, anti apoptosis and anti-inflammation [37-39]	Mice [41-43]	Not mentioned [41,42,44]	Yes	Not mentioned	Intravenous	Positive when delivered at or prior to CPR initiation [41-43]	Survival, neurologic function, neurohistopathology [41-44]
	Open K_ATP_ chanel [35-37]	Pigs [44]	Yes [43]				Neutral when delivered 10 min after CPR [41]	Myocardial function, serum nitrite/nitrate levels and hydrogen peroxide level, cardiac mitochondrial swelling [41]
	Enhance NMDA receptors [38]						Negative [44]	Diffusion weighted imaging and MMP-9 activity [42]
								Cardiac output, heart rate and pulmonary arterial pressure [44]
HBO [52,53]	Attenuation of oxidative and inflammatory injury, inhibition of apoptosis, enhance neurogenesis [47,48]	Dogs [52]	Not mentioned	Yes	Yes [52]	Inhaled	Positive	Neurologic function [52] Neurohistopathology [52,53]
		Rats [53]			Not mentioned [53]			Oxygen extraction ratio and cerebral oxygen delivery/utilization [52]
								Expression of Nogo-A/B, Nogo receptors and RhoA expressions [53]
H_2_[57,58]	Anti-oxidant, anti inflammation and anti-apoptosis [55,56]	Rats [57]	Not mentioned	Yes	Yes	Inhaled [57]	Positive	Survival, neurologic function, neurohistopathology [57,58]
		Rabbit [58]				Intraperitoneal [58]		Myocardial function, cardiomyocyte degeneration, lung edema and systemic inflammatory response [57]
								Plasma 8-OHDG and MDA level [58]

## Pharmaceutical approaches

### Adenosine

Adenosine was proposed in the late 1980’s to be an endogenous neuroprotective molecule with multiple functions [6]. Effects of adenosine and its receptor agonists include reducing the release of excitotoxic neurotransmitters, vasorelaxation, anti-inflammatory effects, reduction of metabolism, scavenging free radicals and moderate reduction of brain temperature [6,7]. Only one pre-clinical study evaluated its treatment effects in the setting of global brain injury associated with CA [8]. In a rat model of CA and CPR, post-ischemic administration of adenosine (7.2 mg/kg) was associated with a transient increase in brain total adenylates, hypothermia, increased post-ischemia brain blood flow and reduced brain edema and delayed CA1 neuronal loss in the hippocampus [8].

In the brain, adenosine acts as a neurotransmitter through activation of four specific G-protein-coupled adenosine (A) receptors: A1, A2A, A2B and A3 receptors [9]. The A1 receptor mediates neuroprotection, mostly by blockade of Ca^2+^ influx, which results in inhibition of glutamate release and reduction of glutamate excitatory effects at a postsynaptic level [10]. Unfortunately, selective activation of A1 receptors as a therapeutic approach for brain injury was hampered by major cardiovascular side effects such as bradycardia and hypotension [6,9]. Given that selective activation of A2A and A3 receptors mediated glutamate release and/or microglia activation and cell death, respectively, development of selective antagonists targeting A2A and A3 may offer a possible treatment for brain disorders via anti-excitoxicity, anti inflammation and anti-apoptosis mechanisms [10,11]. Therapeutic applications of discrete adenosine ligands need to be further explored in the setting of global brain ischemia following CA.

### Growth factors/hormones

Under focal ischemic conditions in the brain, administration of several types of exogenous growth factor/hormones has been shown to be neuroprotective. Limited animal studies evaluated their effects in global brain ischemia secondary to CA. However, the neuroprotective effects were not as robust as that found in cerebral ischemia that does not concurrently involve complete systemic circulatory stasis. Furthermore, a short half-life and a low rate of transport through the blood brain barrier (BBB) could challenge clinical applications of growth factor/hormones for brain protection.

1. Brain-derived neurotrophic factor (BDNF)

BDNF is a neurotrophic factor in the nerve growth factor family. It acts as a potent neuronal survival modulator through the tropomyosin-related kinase receptor type B [12]. The neuroprotective role of BDNF has been shown in ischemic injury including focal brain ischemia, hypoxic-ischemia and global brain ischemia induced by four vessels occlusions [12,13]. The multiple protective roles of BNDF against ischemic brain injury may involve 1) anti-apoptosis by up-regulating B-cell lymphoma-2 (Bcl-2) and inhibiting intracellular calcium overload; 2) anti-inflammation by suppressing pro-inflammatory tumor necrosis factor (TNF)-α expression while increasing anti-inflammatory interleukin (IL)-10 expression; 3) attenuating neurotoxicity mediated by N-methyl-D-aspartate (NMDA) and glutamate; 4) promoting neural regeneration and increasing axonal sprouting and synaptogenesis as well as angiogenesis [12].

However, BDNF failed to provide neurological benefit in a rat model of 6 minute CA and CPR [14]. Neurologic deficits and neurohistopathology at 1, 3 and 7 days post-CA were not different between BDNF treated and placebo treated rats.

2. Insulin-like growth factor-1 (IGF-1) and glycine-proline-glutamate (GPE)

IGF-1 participates in somatic and vascular growth, glucose hemostasis and brain development [15,16]. By binding to its specific receptor and binding proteins, IGF-1 exerts pleiotropic neuroprotection including potent antiapoptotic properties, modulation of BBB permeability and neuronal excitability [17]. Activation of hypoxia-inducible factor (HIF)-1α might also be part of the mechanism underlying IGF-1 promoted cell survival after cerebral ischemia [18]. In a rat model of bilateral carotid occlusion and systemic hypotension, the combination therapy of IGF-1 and hypothermia protected the brain against global ischemic injury. After 8 minutes of untreated global brain ischemia, at the onset of reperfusion one of three treatment regimens was initiated: intravenous (IV) administration of 0.6 U/kg IGF-1 alone; hypothermia (32˚C for 4 h) alone; or combined IGF-1 with hypothermia. Only IGF-1 combined with hypothermia preserved hippocampal CA1 structure and improved neurological function compared to non-treated rats [19].

GPE is a bioactive cleavage product from N-terminal IGF-1 and is also protective against ischemic brain injury [20]. The multiple mechanisms of GPE may be modulation of inflammation, promotion of astrocytosis, inhibition of apoptosis and vascular remodeling [20]. The effects of IGF-1 and GPE were compared in a rat model of CA and CPR [21]. Rats were randomized to receive intracerebroventricular (ICV) IGF-1, GPE, or placebo as a constant infusion for up to 7 days post-ischemia. Neurohistopathologic staining of the CA1 region of the hippocampus was performed at 3, 7 and 14 days. The early significant reduction in the number of apoptotic cells was only observed in the GPE group at 3 days, but this did not persist to 7 or 14 days. From a translational point of view, GPE is a small neuropeptide that can pass BBB easily, thus giving advantages over growth factors in the treatment of brain injury. Nevertheless, it requires a continuous IV infusion to maintain stable central uptake due to the extremely short half-life of GPE [20].

3. Granulocyte colony stimulating factor (G-CSF)

G-CSF is an endogenous peptide hormone of the hematopoietic system that has been evaluated in clinical trials for ischemic stroke [22,23]. By binding to G-CSF receptors in the brain, G-CSF has neuroprotective effects through anti-apoptosis and anti-inflammation as well as through enhancing neurogenesis [24]. A rat model of global brain ischemia induced by 2-vessel occlusion with hemorrhagic hypotension demonstrated the neuroprotective effect of G-CSF [25]. In a rat CA and CPR model, ICV administration of G-CSF (7 or 14 days), however, did not benefit survival, neurobehavioral testing or histopathology over 14 days post-resuscitation [26]. Instead, application of G-CSF increased neuronal damage at 14 days after CA [26].

4. Estrogen

Estrogen can bind to two classic nuclear receptors, namely, estrogen receptor (ER)-α and ER-β, to produce physiologic and neuroprotective effects [27]. A number of studies have demonstrated that estrogen is neuroprotective in brain injury [27,28]. Multiple cellular pathways of cell protection have been associated with estrogen [27]. These involve extracellular signal-regulated kinase/mitogen activated protein kinase signaling and cross talking with IGF-1, which leads to cAMP responsive element binding protein phosphorylation and subsequent neuronal survival and neurogenesis following ischemic insult [29].

Several preclinical studies were performed to evaluate the neuroprotective effect of estrogen against global brain ischemia. In a mouse model of CA (10 minutes) and CPR, different single IV injection dosages of estrogen (0.5, 2.5, 12.5, 25, or 50 micrograms) acutely after resuscitation did not benefit histopathology within hippocampi at 3 days post-resuscitation [30]. But the lowest dose of estrogen resulted in attenuated neuronal injury in the rostral and caudal caudoputamen regions. In a follow-up study, the same group demonstrated the neuroprotection of long-term administration of estrogen, which was most likely mediated by ER-β [31]. They first randomized male mice to receive 17β estradiol (E2, IV loading dose of 0.5 or 2.5 micrograms followed by 12.6 micrograms administered by subcutaneous implant for 3 days) or placebo after 10 minutes of CA followed by CPR. E2 at low or high dose had a neuroprotective effect on the rostral and caudal caudoputamen, but no effects in the hippocampal CA1 region. Neurologic functional recovery was only significant on day 3 with the higher loading dose. Secondly, they tested estrogen receptor (ER) agonist specific for the α or β. The ER-β, but not ER-α, agonist showed improved neuronal survival in striatum and hippocampal CA1 compared to placebo.

Independent of ER-α and-β, G protein-coupled estrogen receptor 1/G-protein-coupled receptor 30 (GPER1/GPR30) is a recently identified membrane receptor that binds estrogen with high affinity [32]. In a mouse model of CA, chronic treatment of male mice with GPR 30 agonist G1 decreased neuronal injury to an extent comparable to estrogen treatment. The increased expression of small-conductance calcium-activated potassium channel 2 (SK2) by G1 could protect neurons from ischemic injury [32]. Other studies also suggest that GPR30 is involved in estrogen neuroprotection through depression of the NMDA receptor 2B (NR2B)-containing NMDA receptors, thus reducing excitoxicity [33,34]. Developing agonists specifically targeting GPR may provide a promising pharmaceutical approach for neuroprotection.

## Gas-mediator approaches

**Figure 1:**
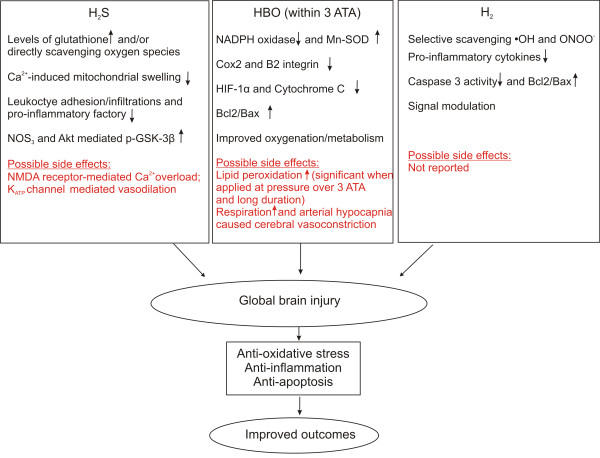
**Neuroprotective mechanisms of gas-mediated approaches after resuscitation from cardiac arrest.** Hydrogen sulfide (H_2_S), hyperbaric oxygen (HBO) and hydrogen gas (H_2_) exert anti-oxidant, anti-inflammation and anti-apoptosis effects through regulation of variable signaling pathways. Such comprehensive brain protection leads to improved outcomes in the setting of global brain injury associated with cardiac arrest.

Gas-mediators exert whole brain protection through comprehensive and complementary mechanisms (Figure 1). Favorable biomembrane permeability renders them attractive alternatives for treatment of global ischemia following CA.

### Hydrogen sulfide

Hydrogen sulfide (H_2_S) was originally known as a toxic gas and environmental pollutant. However, there has been growing interest in the potential role of H_2_S as an endogenous signaling molecule: a third gasotransmitter [35,36]. By opening ATP-sensitive potassium (K_ATP_) channels, endogenous H_2_S acts as a vasorelaxant [35-37]. A number of preclinical studies suggested exogenous low concentration H_2_S protects cells from oxidative stress by increasing levels of glutathione (a cellular major and potent antioxidant) and/or directly scavenging oxygen species [38]. It also exerts anti-apoptotic and anti-inflammation effects [37,39]. In addition, H_2_S facilitates the induction of hippocampal long-term potentiation by enhancing the activity of NMDA receptors [40]. In animal studies of global ischemia brain injury, H_2_S or H_2_S donors provided mixed results. Although H_2_S can protect against inflammation, it may also be involved in intracellular calcium overload and worsen secondary neuronal injury [35,36,39]. These data suggested that H_2_S may have beneficial and/or detrimental effects depending on what pathological state and/or what phase in a certain pathological state it is administered.

### Sodium sulfide, sodium hydrogen sulfide

In mouse model of CA and CPR, administration (IV) of sodium sulfide (Na_2_S, a hydrogen sulfide donor) 0.55 mg/kg at CPR initiation significantly improved survival and neurological deficits at 24 hours [41]. It also significantly decreased apoptosis in the hippocampus, and prevented myocardial dysfunction [41]. Delaying Na_2_S administration to 10 minutes after CPR was also investigated but did not confer the same advantages as when administered at CPR initiation. No formal long-term analysis was performed in this study, but pilot studies show a significant long-term survival advantage in treatment group mice. A similar study investigated the mechanism by which by sodium hydrogen sulfide (NaHS; another hydrogen sulfide donor) conferred benefit [42]. Study methods were similar to Na_2_S studies, but data was collected over 10 days and abnormal water diffusion in the brain was investigated using hyperintense diffusion-weighted imaging. Mice treated with NaHS had significantly improved 10-day survival, neurological outcome, and reduced brain water diffusion. Results differed in a mouse study utilizing a very similar protocol [43]. At CPR initiation, Na_2_S was administered (0.5 mg/kg IV bolus) and continued as an infusion (1 mg/kg/hr) for 6 hours. The only significant difference was better neurological function scoring at 3 days in mice treated with Na_2_S. No other differences were seen between groups with respect to neurological function or neurohistopathology.

In contrast, researchers using a pig model of prolonged CA and CPR reported detrimental effects. Na_2_S (1 mg/kg or 0.3 mg/kg) injection and infusion given at 1 min after starting CPR did not improve initial resuscitation success. It further compromised early postresustication hemodynamics by reducing cardiac output, heart rate and pulmonary arterial pressure [44]. Such deleterious outcome was more significant in the high dose Na_2_S group. H_2_S mediated cerebral ischemia damage has been attributed to increased excitotoxicity through activating NMDA receptors in experimental focal ischemia [45] or due to its vasodilatation effect [44]. These conflicting results may be due to differences in drug administration time between the rodent model (at CPR initiation) and porcine model (1 minute after starting CPR). It suggests that the temporal window of opportunity for improving the outcome of CA/CPR is relatively narrow and may require delivering Na2S immediately after reperfusion. This feature echoes a previous study of ischemic post-conditioning that found the delay of onset of post-conditioning by only 1 minute aborts protection in rabbit heart [46].

### Hyperbaric oxygen (HBO)

HBO at low pressure (within 3 ATA) has been shown to ameliorate brain injury in a variety of animal models including focal cerebral ischemia, neonatal hypoxia–ischemia and subarachnoid hemorrhage [47]. Possible neurotoxicity was only found when HBO was applied at high pressure (>3ATA) and long duration [47]. Adverse effects include increased spontaneous respiratory rate that can lead to arterial hypocapnia with reduced cerebral blood flow, increased lipid peroxidation and seizures [47]. The multiple mechanisms of HBO include enhancing tissue oxygenation/metabolism, reduction of oxidative stress, inflammation modulation, and inhibition of apoptosis as well as enhancing neurogenesis [47,48]. It suggests low pressure of HBO may provide a promising neuroprotective strategy for CA patients favoring neurological function recovery. Neuroprotective effects of HBO therapy have been reported in animal models of global brain ischemia induced by clamping of the ascending aorta and cavae [49], infusing Elliott’s B solution into the subarachnoid space [50] and 4-vessel occlusion [51].

Positive results were consistently observed in two studies using a global brain ischemia model involving whole body ischemia. In a dog model of CA, animals were randomized to control or HBO after CA and resuscitation [52]. HBO was administered at 2.7 ATA for 60 minutes starting 1 hour after ROSC. The functional status of the dogs was significantly better and neuronal damage was significantly less in the HBO group at 1 day after ROSC. Cerebral oxygen extraction ratio was also significantly less in the HBO group. In a rat model of global brain ischemia with hypotension, HBO (3 ATA) applied at 1 h after ischemia for total of 2 h significantly reduced neuronal death in the CA1 region of the hippocampus and the cortex [53].

Additional preclinical studies using clinically relevant CA models are needed to establish the optimal dose and time for treatment as well as the safety of applying HBO treatment following ROSC, particularly when intensive care is required.

### Molecular hydrogen (H_2_)

Molecular hydrogen has previously been proposed as a novel selective hydroxyl radical and peroxynitrite scavenger in the treatment of focal brain ischemia [54]. Emerging evidence has demonstrated its protective a role in a variety of diseases through anti-oxidant, anti-inflammation and anti-apoptotic effects [55,56]. There is no risk of combustion at concentrations less than 4% H_2_[56]. In two animal models of global brain ischemia, hydrogen gas or hydrogen rich saline benefitted overall outcome after CA, including neurological function [57,58]. The protective effects have been shown to be comparable to therapeutic hypothermia [57].

Rats underwent 5 minutes of CA followed by CPR and were randomized to sham, control (ventilation with 98% O_2_ + 2% N_2_), therapeutic hypothermia (TH), hydrogen gas (H_2_, ventilation with 98% O_2_ + 2% H_2_), or TH + H_2_ groups [57]. Twenty-four and seventy-two hour survival was significantly improved in rats treated with H_2_, H_2_ + TH, or TH compared to controls. At 24 and 48 hours, the H_2_, H_2_ + TH, and TH groups were significantly better than the control group, and the H_2_ + TH group was significantly better than the TH and H_2_ groups alone. Systemic inflammatory response measured at 2 hours post ROSC showed significantly reduced levels of IL-6 in H_2_ and H_2_ + TH groups compared to control or TH groups. Other significant effects seen with the H_2_ gas included maintaining low left ventricular end diastolic pressure during the first 2 hours after ROSC when compared to controls, decreased lung edema at 24 hours post-CA (levels same as sham rats), and attenuation of cardiomyocyte degeneration.

In rabbits subjected to CA followed by CPR, intraperitoneal injection of H_2_ rich saline or placebo was done at CPR initiation [58]. Animals were assigned to sham (no CA); CA; CA + low dose H_2_ (10 ml/kg; CA + H_2_ group 1); and CA + high dose H_2_ rich saline (20 ml/kg; CA + H_2_ group 2). Intraperitoneal H_2_ rich saline (low and high dose) significantly improved 72-hour survival, reduced neuronal injury, and reduced plasma oxidative markers. Neurologic functional recovery was significantly improved only with 20 ml/kg H_2_ compared to controls at 72 hours.

H_2_ is cost-effective and has low toxicity with little drug-drug interaction. These features could make H_2_ administration an ideal neuroprotection approach that could be implemented during CPR and/or after ROSC.

## Conclusions

Comprehensive neuroprotective strategies benefit global brain ischemia associated with cardiac arrest by targeting oxidative stress, cell apoptosis and inflammatory responses. Based on limited preclinical findings, gas-mediator strategies, particularly, HBO (within 3 ATA) and molecular H_2_, have shown promise for improving survival and neurologic outcomes when implemented either during or immediately after CPR. Such comprehensive brain protective therapies may provide advantages over selective treatment strategies that have been developed for global brain ischemia over the past few decades. To date, no clinical studies have been conducted to test these approaches in patients with global brain ischemia. Translational research is needed to further elucidate fundamental neuroprotective mechanisms and effective treatment protocols prior to clinical applications in the future.

## Abbreviations

AHA: American Heart Association; BBB: Blood brain barrier; Bcl-2: B-cell lymphoma-2; BDNF: Brain-derived neurotrophic factor; CA: Cardiac arrest; CPR: Cardiopulmonary resuscitation; ER: Estrogen receptor; G-CSF: Granulocyte colony stimulating factor; GPE: Glycine-proline-glutamate; GPER: G protein-coupled estrogen receptor; GPR: G-protein-coupled receptor; H2: Molecular hydrogen; H2S: Hydrogen sulfide; HBO: Hyperbaric oxygen; HIF: Hypoxia-inducible factor; ICV: Intracerebroventricular; IGF-1: Insulin-like growth factor-1; IL: Interleukin; IV: Intravenous; KATP: ATP-sensitive potassium; Na2S: Sodium sulfide; NaHS: Sodium hydrogen sulfide; NMDA: N-methyl-D-aspartate; NR2B: NMDA receptor 2B; ROSC: Restoration of spontaneous circulation; TH: Therapeutic hypothermia; TNF: Tumor necrosis factor.

## Competing interests

The authors declare that they have no competing interest.

## Authors’ contributions

LH participated in design, literature search, evaluation of papers for inclusion and writing of this review. PA participated in evaluation of papers for inclusion and writing of this review. JG participated in literature search, evaluation of papers for inclusion and revision of this review. DM participated in literature search, evaluation of papers for inclusion and writing this review. JZ participated in the design and revision of this review. RA participated in design, literature search, evaluation of papers for inclusion and writing of this review. All authors read and approved the final manuscript.
